# Clinically differential diagnosis of human granulocytic anaplasmosis and severe fever with thrombocytopenia syndrome

**DOI:** 10.1038/s41598-023-32061-1

**Published:** 2023-04-26

**Authors:** Dong-Min Kim, Byung Jun Yu, Da Young Kim, Jun-Won Seo, Na-Ra Yun, Choon Mee Kim, Young Keun Kim, Sook In Jung, Uh Jin Kim, Seong Eun Kim, Hyun ah Kim, Eu Suk Kim, Jian Hur, Sun Hee Lee, Hye Won Jeong, Jung Yeon Heo, Dong Sik Jung, Jieun Kim, Sun Hee Park, Yee Gyung Kwak, Sujin Lee, Seungjin Lim, Shilpa Chatterjee

**Affiliations:** 1 Department of Internal Medicine, College of Medicine, Chosun University, Gwangju, Republic of Korea; 2 Premedical Science, College of Medicine, Chosun University, Gwangju, Republic of Korea; 3 Department of Internal Medicine, Wonju College of Medicine, Yonsei University, Wonju, Republic of Korea; 4 Department of Infectious Diseases, Chonnam National University Medical School, Gwangju, Republic of Korea; 5 Division of Infectious Diseases, Keimyung University Dongsan Hospitial, Keimyung University School of Medicine, Daegu, Republic of Korea; 6 Department of Internal Medicine, Seoul National University Bundang Hospital, Seoul National University College of Medicine, Seongnam, Republic of Korea; 7 Department of Internal Medicine, Yeungnam University Medical Center, Daegu, Republic of Korea; 8 Department of Internal Medicine, School of Medicine, Pusan National University, Busan, Republic of Korea; 9 Department of Internal Medicine, College of Medicine, Chungbuk National University, Cheongju, Republic of Korea; 10 Department of Infectious Diseases, School of Medicine, Ajou University, Suwon, Republic of Korea; 11 Department of Internal Medicine, College of Medicine, Dong-A University, Busan, Republic of Korea; 12 Department of Internal Medicine, College of Medicine, Hanyang University, Seoul, Republic of Korea; 13 Division of Infectious Diseases, Department of Internal Medicine, College of Medicine, The Catholic University of Korea, Seoul, Republic of Korea; 14 Department of Internal Medicine, Inje University Ilsan Paik Hospital, Goyang, Republic of Korea; 15 Department of Internal Medicine, College of Medicine, Pusan National University, Yangsan, Republic of Korea; 16 Department of Biomedical Science, College of Medicine, Chosun University, Gwangju, Republic of Korea

**Keywords:** Bacterial infection, Viral infection

## Abstract

This study analyzed HGA and SFTS in patients with suspected tick-borne infection by focusing on key differences that clinicians can easily recognize. A retrospective analysis was performed on confirmed patients with HGA or SFTS in 21 Korean hospitals from 2013 to 2020. A scoring system was developed by multivariate regression analysis and accuracy assessment of clinically easily discriminable parameters was performed. The multivariate logistic regression analysis revealed that sex (especially male sex) (odds ratio [OR] 11.45, P = 0.012), neutropenia (< 1500) (OR 41.64, P < 0.001), prolonged activated partial thromboplastin time (OR 80.133, P < 0.001), and normal C-reactive protein concentration (≤ 1.0 mg/dL; OR 166.855, P = 0.001) were significantly associated with SFTS but not with HGA. Each factor, such as meaningful variables, was given 1 point, and a receiver-operating characteristic curve with a cutoff value (> 1) in a 5-point scoring system (0–4 points) was analyzed to evaluate the accuracy of differentiation between HGA and SFTS. The system showed 94.5% sensitivity, 92.6% specificity, and an area under the receiver-operating characteristic curve of 0.971 (0.949–0.9). Where HGA and SFTS are endemic, the scoring system based on these four parameters such as sex, neutrophil count, activated partial thromboplastin time, and C-reactive protein concentration will facilitate the differential diagnosis of HGA and SFTS in the emergency room in patients with suspected tick-borne infectious diseases.

## Introduction

Human granulocytic anaplasmosis (HGA) and severe fever with thrombocytopenia syndrome (SFTS) are tick-borne infectious diseases in humans caused by *Anaplasma phagocytophilum* and SFTS virus (SFTSV) (which belongs to the *Bandavirus* genus of the *Bunyavirales* order, *Phenuiviridae* family), respectively^1,2^.

HGA and SFTS are the most common tick-borne infectious diseases in Korea, China, and Japan^3^. SFTS has also been detected based on molecular and serological tests in both humans and animals from Vietnam, Taiwan, Thailand, and Myanmar^4–7^. *Haemaphysalis longicornis* is the main vector of SFTS. *Amblyomma testudinarium* and *Ixodes nipponensis* are also considered possible vectors of SFTS.

Ticks are the vector of HGA, in which *Ixodes scapularis* is the main vector in the northeastern and the upper midwest regions of the United States; in contrast, *Ixodes pacificus* is the main vector of HGA in the northern Pacific coast^8^. In Korea, *A. phagocytophilum* has been detected in ticks, including *Haemaphysalis longicornis*, *Ixodes nipponensis*, and *Ixodes persulcatus*^9^, and the first case of HGA was reported in 2014^10^.

In a Chinese study on patients with an acute febrile disease in a surveillance program in 2009, pathogens were not detectable in patients with symptoms similar to those of HGA, in which cell culture, electron microscopy, and nucleic acid sequencing detected SFTSV in blood samples from these patients for the first time^11,12^.

In the U.S.A., 2 patients with symptoms similar to SFTS have been reported in northwestern Missouri; of these 2 patients, the Heartland virus was 70% genetically identical to SFTSV^13,14^.

Since 2018, the number of SFTS cases in Korea has increased steadily (259 cases in 2018) but lacks effective treatment^15^. In contrast, HGA which has been described as an emerging infectious disease in Korea, can be treated with tetracycline-based antibiotics^10^. Typically, HGA and SFTS both are caused by tick bites in patients who are active outdoors with similar early clinical manifestations upon admission. The two diseases also have a similar epidemic season, which makes differentiating the diagnosis of these diseases challenging.

Although HGA and SFTS differ significantly in terms of transmission (there is a lack of human-to-human transmission in HGA, while SFTS can get transmitted by needle stick injury and cardiopulmonary resuscitation), features that can aid the clinical differentiation between the two are lacking, which creates the need for rapid clinical discrimination of the two diseases and prompt administration of effective antibiotics^16^. However, till date no study has compared the clinical features of HGA and SFTS, thus our current study analyzed the important differences between HGA and SFTS in patients with suspected tick-borne infectious diseases, in which our findings should be useful for clinicians.

## Methods

### Patients

This study retrospectively analyzed the data from a study that was conducted using a pre-written case report form. The present study was approved by the Institutional Review Boards (IRBs) of Chosun University Hospital and participating institutions and all research was performed in accordance with relevant guidelines/regulations.

The data on 221 patients who received a diagnosis of SFTS at 21 hospitals in Korea and 33 patients who received a diagnosis of HGA at the Chosun University Hospital from 2013 to 2020 were analyzed and compared^17,20^.

HGA was diagnosed as follows: (1) successful culture of *A. phagocytophilum*; (2) more than a four-fold increase in the antibody concentrations in the indirect fluorescent antibody (IFA) test^13^ during the acute and convalescent phases; (3) the presence of more than two specific genes of *A. phagocytophilum* according to polymerase chain reaction (PCR) test results; and (4) the presence of more than one specific gene of *A. phagocytophilum* and antibody titers higher than 1:16 or 1:80 for IFA IgM and IgG, respectively. The PCR based HGA diagnosis was performed targeting genes specific for *A. phagocytophilum* including the GroEL heat-shock protein gene (*groEL*), the ankyrin-repeat protein AnkA gene (*ankA*), and the major surface protein 2 gene (*msp2*). The details of the PCR and IFA protocols are described in Refs.^17–19^.

SFTS was diagnosed using the following criteria: positivity for the SFTSV target gene in conventional, nested, and real-time PCR tests or more than a four-fold increase in the IFA antibody level^20^.

### Statistical analysis

The Chi-square or Fisher’s exact tests were performed to compare the variables. The *t* test was conducted to compare continuous variables. Logistic regression analysis was performed to develop a scoring system that could discriminate between HGA and SFTS. The Statistical Package for the Social Sciences (SPSS), version 22.0 (IBM Corp., Armonk, NY; http://www.ibm.com/analytics/spss-statistics-software; 2013), and MedCalc, version 18.11.6 (MedCalc Software Ltd, Ostend, Belgium; https://www.medcalc.org; 2019), were used for statistical analyses.

### Consent to publish

Written informed consent was obtained from all the patients for participating in the study.

## Results

### Diagnostic tests

Among the patients who visited our hospitals between 2013 to 2020, PCR tests were performed for suspected patients of having tick-borne infectious diseases. The average duration of illness at the time of the diagnostic testing of the patients who visited the hospital was 4.67 ± 4.955 (mean ± SD) days. Among 33 HGA positive patients, 28 (28/33, 84.84%) were positive for two or more genes for *A. phagocytophilum*, and 5 (5/33, 15.16%) were positive with a four-fold increase in antibody titer between the acute and recovery phases. A total of 33 patients were confirmed to have HGA. To exclude the effects of other diseases, all patients tested positive for any other pathogen besides HGA were excluded. Additionally, all the 33 HGA positive cases were negative for both SFTS PCR and antibody testing.

Similarly, among 221 SFTS positive patients, 203 patients had a confirmed diagnosis of SFTS between 2013 and 2020 by SFTS-specific conventional, N-PCR targeting S-segment and real-time PCR test, and 18 of them were also diagnosed via more than four-fold IgG antibody increase using IFA. Moreover, we performed the three multiplex real-time PCR targeting scrub typhus, leptospira, and hemorrhagic fever with the renal syndrome to confirm the PCR positivity. We excluded cases that showed any evidence of positive PCR results other than those for SFTS and anaplasmosis.

### Clinical characteristics

The clinical characteristics of 33 patients with HGA and 221 patients with SFTS are shown in supplementary table S1. In Korea, SFTS was found to occur between the spring and autumn. By contrast, HGA mainly manifested in spring and summer between March and August in approximately 85% of the patients, in which 63.6% of the patients with HGA and 30.8% of the patients with SFTS were farmers. Further detailed analysis of the clinical symptoms showed that fever, hemorrhagic symptoms, central-nervous-system symptoms, and expectoration were more common in patients with SFTS than those patients with HGA. In contrast, fatigue and thirst were more common in patients with HGA than those patients with SFTS. Intensive care unit (ICU) admission rates and mortality rates were high in patients with SFTS. In contrast, the patients with HGA were admitted to the ICU, but no deaths occurred.

### Laboratory findings

A comparison of the laboratory findings between the 2 patient groups revealed that the white blood cell (WBC/µL) count was 2369.95 ± 2464.13 in the SFTS group and 4780.3 ± 2874.07 in the HGA group, suggesting that patients with SFTS had significant leukocytopenia. In particular, absolute neutrophil and lymphocyte counts of less than 1500 are more common in patients with SFTS than those patients with HGA (Supplementary Table S2). The WBC count was less than 3000 in 86.4% of patients with SFTS but only 31% in patients with HGA. Among the patients with SFTS, 53.8% had a platelet count of less than 70,000, whereas only 24.2% in patients with HGA. The proportion of the patients with a platelet count of less than 70,000 significantly differed between the two patient groups.

Aspartate aminotransaminase (AST) activity in the blood was 288.99 ± 473.04 in patients with SFTS, whereas 123.52 ± 146.2 in patients with HGA. aPTT was 48 ± 39.79 and 30.95 ± 4.81 s in patients with SFTS and HGA, respectively. C-reactive protein (CRP) concentration was 1.84 ± 6.38 in patients with SFTS (viral disease), whereas 8.49 ± 7.24 in patients with HGA, in which this difference between the 2 groups was significant.

### Statistical analysis

Variables that were significantly different between the patients with SFTS and HGA, which were helpful for differential diagnosis, were included in a univariate regression analysis. The results are presented in Supplementary Table S3.

**Figure 1:**
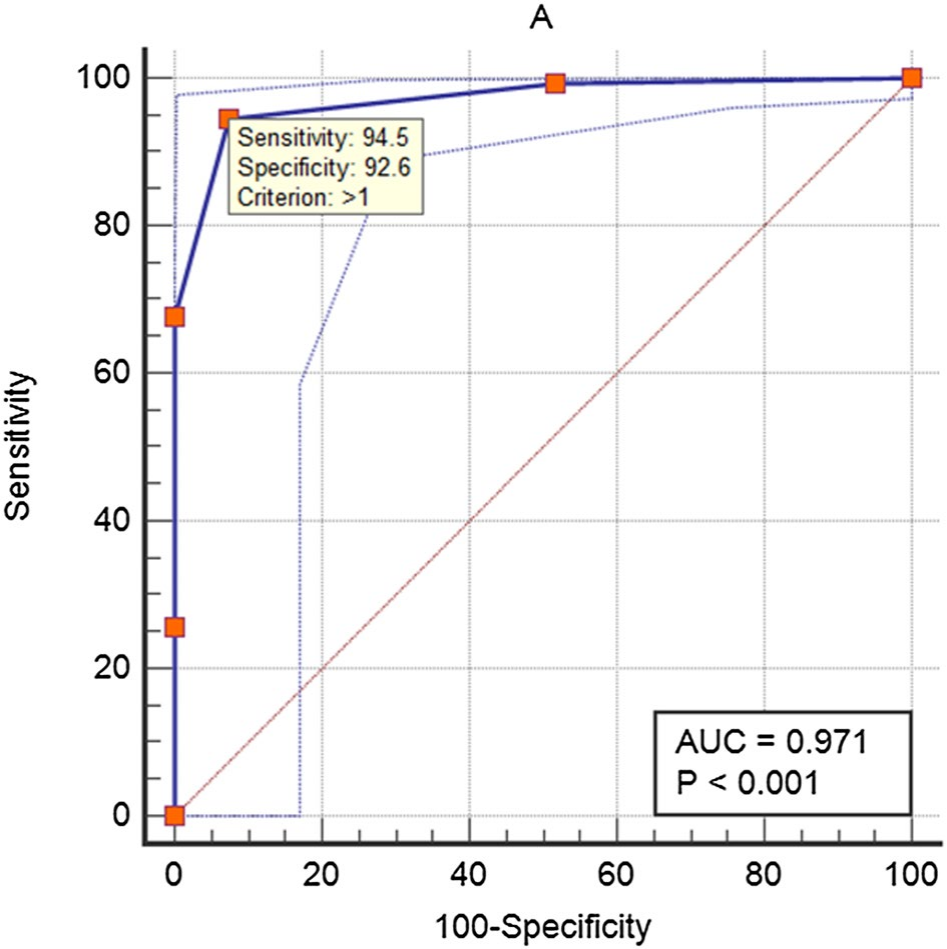
Evaluation of the area under the receiver-operating characteristic (ROC) curve (AUC) for a total score of > 1 in a classification model with a score of 1 for male, neutropenia (< 1500), aPTT prolongation (> 35 s), and normal C-reactive protein concentration (≤ 1.0 mg/dL) as factors among 33 patients with HGA and 221 patients with SFTS.

Multivariate logistic regression analysis was conducted next to differentiate between HGA and SFTS. Sex, especially the male (odds ratio [OR] 11.45, P = 0.012), neutropenia (< 1500) (OR 41.64, P ≤ 0.001), aPTT prolongation (> 35 s) (OR 80.133, P ≤ 0.001), and normal CRP concentration (≤ 1.0 mg/dL; OR 166.855, P = 0.001) were significantly associated with SFTS but not HGA. A score of 1 is assigned to each of these significant factors, and the appropriate cutoff value of the receiver-operating characteristic (ROC) curve is > 1 in a scoring system with a total score from 0 to 4 points (Supplementary Table S4). A total score of > 1 was used to assess the accuracy of the discrimination between HGA and SFTS. Sensitivity, specificity, and the area under the ROC curve (AUC) for the prediction of SFTS are 94.5%, 92.6%, and 0.971 (0.949–0.989), respectively (Fig. 1).

## Discussion

In 2009, patients with fever, leukocytopenia, thrombocytopenia, and gastrointestinal symptoms were noted in the Hubei and Henan provinces of China. The aforementioned symptoms are very similar to the clinical symptoms of HGA. Studies have tested whether the disease is caused by *A. phagocytophilum* or other pathogens. No *A. phagocytophilum* deoxyribonucleic acid (DNA) or antibody reactions were observed. Instead, SFTSV, a novel virus, was detected^11^. SFTS was initially reported mainly in China, Japan, and Korea. Common mite-borne and tick-borne diseases in these three countries include scrub typhus and HGA.

Scrub typhus and rickettsial infections have characteristic features of eschar, which is an erythematous ring with scales around a black scab, showing a clear difference from a simple tick bite site. Nevertheless, rash and eschar are rarely observed in patients with SFTS and HGA, in whom these diseases also have similar clinical symptoms. Therefore, differentiating between HGA and SFTS using symptoms or laboratory findings is challenging^21^. Previously, study performed by Wormser stated that clinical laboratory features and nosocomial transmission characteristics of 9 cases reported as HGA in China in 2008 were significantly different from the cases of HGA reported in the U.S.A., suggesting that SFTS may have been incorrectly reported as HGA^22^.

In cases of non-specific febrile illness of unknown origin, maintaining a high level of clinical suspicion for anaplasmosis or other tick-borne infections, especially during the spring and summer months when ticks are most active, is strongly recommended. To avoid serious illness, symptoms must be recognized and treated early. Patient survival depends on quickly suspecting and diagnosing SFTS based on clinical characteristics, laboratory findings, and suitable epidemiological studies at the time of patient admission. Moreover, patients should be treated presumptively depending on the clinical suspicion because anaplasmosis can be difficult to diagnose, especially in its early stages. Despite the difficulties, *A. phagocytophilum* culture isolation as a diagnostic test should be carried out because conventional hospital blood cultures cannot detect the organism.

In this study, we attempted to identify the variables that could be objectively evaluated; predicting SFTS by means of a total score of > 1 showed a sensitivity of 94.5% and a specificity of 92.6% with an AUC of 0.971. These parameters can be easily tested in the emergency room within a few hours. Thus, the proposed scoring system may be employed for easy differentiation between HGA and SFTS in endemic regions of these diseases.

In this retrospective study, a classification model was devised to differentiate between HGA and SFTS. On the other hand, the model was not validated properly. Therefore, prospective studies are necessary to validate this model in the future.

In conclusion, parameters such as sex, neutropenia, aPTT prolongation, and normal CRP levels were identified, which can be easily assessed in the emergency room. Consequently, the scoring system based on these four variables selected in this study will facilitate the differential diagnosis of HGA and SFTS in patients with suspected tick-borne infectious diseases in endemic regions of HGA and SFTS.

## Supplementary Information

Supplementary Tables.

## Data Availability

We shared our data to figshare with 10.6084/m9.figshare.20461980.
