# Supplementary material for: Clinically differential diagnosis of human granulocytic anaplasmosis and severe fever with thrombocytopenia syndrome
Source: Sci Rep. 2023 Apr 26;13:6837. doi: 10.1038/s41598-023-32061-1 (PMC10133271; doi:10.1038/s41598-023-32061-1)
Supplement: Supplementary file 1 — Supplementary Tables. [file 41598_2023_32061_MOESM1_ESM.docx]

Supplementary Table S1. Clinical characteristics of human granulocytic anaplasmosis (HGA) and severe fever with thrombocytopenia syndrome (SFTS)

| Variable | SFTS (n=221)  Mean±SD  or N (%) | HGA (n=33)  Mean±SD  or N (%) | *P* value |
| --- | --- | --- | --- |
| Age, years | 65.95±13.44 | 71.18±12.16 | .036 |
| Sex (male) | 115 (52) | 10 (30.3) | .020 |
| Comorbidity | 135 (61.1) | 22 (66.7) | 0.538 |
| Diabetes | 46 (20.8) | 1 (3) | .014 |
| Hypertension | 86 (38.9) | 8 (24.2) | 0.103 |
| Cardiovascular disease | 13 (5.9) | 1 (3) | 0.503 |
| Congestive heart failure | 4 (1.8) | 0 | 0.436 |
| Chronic liver disease | 8 (4) | 7 (21.2) | <.001 |
| Asthma | 2 (0.9) | 0 | 0.583 |
| COPD | 3 (1.4) | 0 | 0.501 |
| Solid tumor | 5 (2.3) | 0 | 0.383 |
| Season |  |  | .001 |
| Spring-summer (March-August) | 122 (55.2) | 28 (84.8) |  |
| Autumn-winter (September-February) | 99 (44.8) | 5 (15.2) |  |
| Geographic location (where infected) |  |  | .019 |
| Metropolitan area | 9 (4.1) | 4 (14.8) |  |
| Province | 209 (95.9) | 23 (85.2) |  |
| Occupation: agriculture | 68 (30.8) | 21 (63.6) | <.001 |
| Clinical symptoms |  |  |  |
| Fever | 208 (94.1) | 22 (66.7) | <.001 |
| Myalgia | 106 (48) | 11 (33.3) | 0.116 |
| Fatigue | 67 (30.7) | 21 (63.6) | <.001 |
| Sore throat | 11 (5.3) | 4 (12.1) | 0.129 |
| Thirst | 19 (8.6) | 7 (21.2) | .038 |
| Expectoration | 37 (17.5) | 1 (3) | .033 |
| GI total symptoms | 168 (76.4) | 20 (60.6) | 0.053 |
| Hemorrhagic symptoms | 39 (17.6) | 1 (3) | .032 |
| Headache | 82 (37.8) | 9 (28.1) | 0.289 |
| Altered mental status | 90 (41.7) | 6 (18.2) | 0.010 |
| CNS total symptoms | 152 (69.1) | 15 (45.5) | .008 |
| Rash | 37 (17.2) | 3 (9.1) | 0.238 |
| Tick bite or eschar | 72 (32.7) | 14 (43.8) | 0.421 |
| ICU admission | 85 (38.6) | 5 (15.2) | .009 |
| In-hospital death | 48 (21.7) | 0 | .003 |

Abbreviations: CNS, central nervous system; COPD, chronic obstructive pulmonary disease; GI, gastrointestinal; ICU, intensive care unit. **GI total symptoms:** anorexia, nausea, vomiting, diarrhea, dyspepsia, flank pain, abdominal pain, abdominal tenderness**. Hemorrhagic symptoms:** epistaxis, gingival bleeding, hemoptysis, GI bleeding, hematemesis/melena, gross hematuria, purpura, petechiae**. CNS total symptoms:** headache, dizziness, seizure, neck stiffness, altered mental state

**Supplementary Table S2. Laboratory findings at the initial presentation**

| Laboratory findings | SFTS (n=221)  Mean±SD  or N (%) | HGA (n=33)  Mean±SD  or N (%) | *P* value |
| --- | --- | --- | --- |
| White blood cell count/ µL | 2369.95±2464.13 | 4780.3±2874.07 | <.001 |
| Leukopenia (<5000) | 208 (94.1) | 19 (57.6) | <.001 |
| Leukopenia (<4000) | 201 (91) | 17 (51.5) | <.001 |
| Leukopenia (<3000) | 191 (86.4) | 11 (33.3) | <.001 |
| Neutropenia (<1500) | 151 (70.9) | 3 (9.1) | <.001 |
| Lymphopenia (<1500) | 208 (97.2) | 27 (81.8) | <.001 |
| Hemoglobin | 13.6±1.72 | 12.99±1.46 | 0.054 |
| Platelet count | 76.31±41.1 | 114.7±72.46 | .005 |
| Thrombocytopenia (<150,000) | 210 (95) | 26 (78.8) | .001 |
| Thrombocytopenia (<100,000) | 169 (76.5) | 18 (54.5) | .008 |
| Thrombocytopenia (<70,000) | 119 (53.8) | 8 (24.2) | .002 |
| Thrombocytopenia (<50,000) | 63 (28.5) | 5 (15.2) | 0.106 |
| Total bilirubin | 0.523±0.511 | 0.639±0.261 | 0.456 |
| AST | 288.99±473.04 | 123.52±146.2 | <.001 |
| AST >200 | 69 (31.7) | 3 (9.1) | .008 |
| AST >100 | 129 (59.2) | 12 (36.4) | .014 |
| ALT | 102.57±126.89 | 71.85±81.35 | 0.179 |
| ALT >200 | 23 (10.6) | 1 (3) | 0.171 |
| INR>1.3 | 9 (4.8) | 1 (3) | 0.720 |
| aPTT | 48±39.79 | 30.95±4.81 | .025 |
| aPTT >40 | 105 (54.4) | 1 (3.4) | <.001 |
| aPTT >35 | 150 (77.7) | 4 (14.3) | <.001 |
| CRP | 1.84±6.38 | 8.49±7.24 | <.001 |
| CRP <4 | 186 (91.2) | 8 (26.7) | <.001 |
| CRP <3 | 182 (89.2) | 6 (20) | <.001 |
| CRP <2 | 172 (84.3) | 5 (16.7) | <.001 |
| CK | 1722.87±4434.12 | 949.3±3317.01 | 0.367 |
| CK >300 | 103 (69.1) | 11 (36.7) | .001 |
| CK >200 | 115 (77.2) | 14 (46.7) | .001 |
| LDH >300 | 133 (84.2) | 23 (95.8) | 0.128 |

Abbreviations: ALT, alanine aminotransferase; aPTT, activated partial thromboplastin time; AST, aspartate aminotransferase; CK, creatine kinase; CRP, C-reactive protein; HGA, human granulocytic anaplasmosis; INR, international normalized ratio; LDH, lactate dehydrogenase; SFTS, severe fever with thrombocytopenia syndrome

**Supplementary Table S3. Univariate or multivariate logistic regression analysis of parameters predictive of SFTS**

|  | Univariate | | Multivariate | |
| --- | --- | --- | --- | --- |
|  | Odds ratio (95% CI) | *P* value | Odds ratio (95% CI) | *P* value |
| Sex (male) | 2.495 (1.135-5.487) | 0.023 | 17.314 (1.650-181.680) | 0.017 |
| Season (March-August) | 0.220 (0.082-0.591) | 0.003 | 0.272 (0.031-2.427) | 0.244 |
| Geographic location (Infected area): Province | 4.039 (1.152-14.153) | 0.029 | 1.342 (0.075-24.170) | 0.842 |
| Leukopenia (<4000) | 9.459 (4.154-21.540) | <0.001 | 5.316 (0.724-39.031) | 0.100 |
| Neutropenia (<1500) | 24.355 (7.168-82.749) | <0.001 | 19.635 (1.535-251.087) | 0.022 |
| Thrombocytopenia (<70,000) | 3.646 (1.576-8.436) | 0.003 | 1.03 (0.087-12.268) | 0.980 |
| AST>200 | 4.631 (1.366-15.696) | 0.014 | 0.874 (0.065-11.665) | 0.919 |
| aPTT>35 | 20.93 (6.888-63.603) | <0.001 | 23.953 (1.989-288.479) | 0.012 |
| CRP<2 | 28.417 (11.069-72.95) | <0.001 | 65.638 (6.315-682.257) | <0.001 |

**Supplementary Table S4. Diagnostic performance of the proposed clinical scoring system to differentiate human granulocytic anaplasmosis (HGA) from severe fever with thrombocytopenia syndrome (SFTS)**

| Clinical score^*^  (n=210) | SFTS  (n=183) | HGA  (n=27) | Sensitivity  (95% CI) | Specificity  (95% CI) | Positive likelihood ratio  (95% CI) | Negative likelihood ratio  (95% CI) |
| --- | --- | --- | --- | --- | --- | --- |
| >0 | 182 | 14 | 99.45 (97-100) | 48.15 (28.47-68.1) | 1.92 (1.3-2.8) | 0.011 (0.002-0.08) |
| >1 | 173 | 2 | 94.54 (90.2-97.3) | 92.59 (75.7-99.1) | 12.76 (3.4-48.5) | 0.059 (0.03-0.1) |
| >2 | 124 | 0 | 67.76 (60.5-74.5) | 100 (87.2-100) | NA | 0.32 (0.3-0.4) |
| >3 | 47 | 0 | 25.68 (19.5-32.6) | 100 (87.2-100) | NA | 0.74 (0.47-0.8) |

Abbreviations: CI, confidence interval

^*^Scoring system = (1 × male) + (1 × neutropenia <1500) + (1 × aPTT >35) + (1 × CRP <2)
